# Clinical profile, common thrombophilia markers and risk factors in 85 young Indian patients with arterial thrombosis

**DOI:** 10.1590/1516-3180.2013.1316369

**Published:** 2013-12-01

**Authors:** Mahendra Narain Mishra, Ravi Kalra, Shalesh Rohatgi

**Affiliations:** I MD. Specialist in Pathology and Immunopathology, Department of Pathology, Dr. Lal Path Labs Pvt Ltd., New Delhi, India; II MD, PhD. Department of Cardiology, Indian Naval Hospital Ship Asvini, Colaba, Mumbai, Maharashtra, India; III MD, PhD. Department of Neurology, Command Hospital (WC), Chandimandir, Haryana, India

**Keywords:** Thrombophilia, Myocardial infarction, Stroke, Fibrinogen, Lipoprotein(a), Trombofilia, Infarto do miocárdio, Acidente vascular cerebral, Fibrinogênio, Lipoproteína(a)

## Abstract

**CONTEXT AND OBJECTIVE:**

Arterial thrombosis may occur consequent to hereditary thrombophilia and increased lipoprotein(a) [Lp(a)] and fibrinogen. Our aim was to study the prevalence of common thrombophilia markers in 85 consecutive cases of arterial thrombosis.

**DESIGN AND SETTING:**

A retrospective study was conducted from 85 consecutive young patients treated as outpatients or admitted due to stroke or myocardial infarction at a tertiary care hospital.

**METHODS:**

Eighty-five Indian patients (age < 45 years) presenting ischemic stroke (n = 48) or myocardial infarction (n = 37) and 50 controls were studied for seven thrombophilia markers including antithrombin (AT), factor V, protein C, protein S, activated protein C resistance (APC-R), fibrinogen and Lp(a). Functional assays for protein C, protein S, factor V and APC-R were performed using clotting-based methods. Semi-quantitative estimation of fibrinogen was done using Clauss's method and Lp(a) using immunoturbidimetry. Statistical analysis was done using the Epi Info 6 software.

**RESULTS:**

Thirty-three samples (38.8%) tested positive for one or more thrombophilia markers. The three commonest abnormalities were elevated Lp(a) (20%), fibrinogen (17.6%) and low APC-R (14.2%). Low levels of protein C, protein S and AT were present in 4.7, 9.4 and 7% of the patients, respectively. Overall, the risk factor profile was: smoking (33%), positive family history (15.3%), hyperlipidemia (7%), hypertension, diabetes mellitus and obesity (2.3% each).

**CONCLUSIONS:**

An association was found between low levels of protein C, protein S and AT and arterial thrombosis, but only elevated fibrinogen levels, smoking, positive family history and hyperlipidemia showed statistical significance.

## Abstract (pt)

**CONTEXTO E OBJETIVO:**

Trombose arterial pode ocorrer em consequência de trombofilias hereditárias e de lipoproteína (a) [Lp (a)] e fibrinogênio aumentados. Nosso objetivo foi estudar a predominância de marcadores comuns da trombofilia em 85 casos consecutivos de trombose arterial.

**TIPO DE ESTUDO E LOCAL:**

Um estudo retrospectivo foi realizado sobre 85 pacientes jovens tratados consecutivamente no ambulatório ou admitidos por infarto do miocárdio ou acidente vascular cerebral (AVC) num hospital de cuidado terciário.

**MÉTODOS:**

Oitenta e cinco pacientes indianos (idade < 45 anos) que se apresentaram com AVC isquêmico (n = 48) ou infarto do miocárdio (n = 37) e 50 controles foram estudados para sete marcadores de trombofilia que incluíram antitrombina (AT), fator V, proteína C, proteína S, resistência ativada da proteína C (APC-R), fibrinogênio e Lp (a). Os ensaios funcionais da proteína C, proteína S, fator V e APC-R foram executados por métodos baseados em coagulação. A avaliação semiquantitativa do fibrinogênio foi feita pelo método de Clauss e a Lp(a) por imunoturbimetria. A análise estatística foi feita pelo *software* Epi Info 6.

**RESULTADOS:**

Trinta e três amostras (38.8%) foram positivas para um ou vários marcadores do trombofilia. As anomalias mais comuns foram Lp (a) (20%), fibrinogênio (17.6%) e APC-R (14.2%) elevados. Baixos níveis da proteína C, proteína S e AT foram detectados em 4.7%, 9.4% e 7% dos pacientes, respectivamente. Globalmente, os perfis dos fatores de risco foram: fumo (33%), antecedentes familiares positivos (15.3%), hiperlipidemia (7%), hipertensão, diabetes mellitus e obesidade (2.3% cada).

**CONCLUSÕES:**

Uma associação foi encontrada entre baixos níveis de proteína C, proteína S, AT e trombose arterial, mas somente os níveis de fibrinogênio elevado, tabagismo, história familiar positiva e hiperlipidemia assumiram significância estatística.

## INTRODUCTION

Inherited thrombophilia is more commonly associated with venous rather than arterial thrombosis. There is controversy regarding the role of inherited causes of thrombophilia such as deficiencies of natural anticoagulants (antithrombin, protein C and protein S) and activated protein C resistance (APC-R) in arterial thrombosis, while increased fibrinogen levels and acquired causes such as antiphospholipid antibodies have been implicated. Inherited causes of thrombophilia, especially protein C deficiency, are more common in children with a first arterial ischemic stroke than in healthy children.^1^ The likelihood of detecting at least one thrombophilia marker in young patients with myocardial infarction (MI) who have fewer conventional risk factors is significantly high.^2^

## OBJECTIVE

This study was carried out to study the frequency of multiple thrombophilia markers including APC-R, antithrombin (AT), factor V, protein C, protein S and fibrinogen in patients with primary arterial thrombosis presenting clinically as MI or stroke, since there are very few Indian studies on the subject. 

## METHODS

We conducted a retrospective study in which data pertaining to 85 consecutive cases (48 with ischemic stroke and 37 with MI) and 50 controls were analyzed for the presence of seven thrombophilia markers. The inclusion criteria for patients were the presence of one or more of the following: (i) Age < 45 years at onset of thrombotic episode and (ii) absence of any obvious cause such as prolonged immobilization, sickle cell disease, cardiac arrhythmias or source of thrombus that predisposed towards thrombosis. The control population included age and sex-matched healthy blood donors and volunteers, except for some very young pediatric patients. Informed consent from all participants and approval from the Indian Naval Hospital Ship Asvini Ethics Committee were obtained for this study. Two patients with multiple episodes at the time of recruitment for the study were over 45 years old, but they had had the first episode before reaching 45 years of age and hence were included. 

Factor V levels were estimated, given that very low factor V may cause increased APC-R.^3^ Lp(a) was included in this study because it is an independent risk factor for myocardial infarction in adults and for strokes in children.^4^ ^,^ ^5^ Histories of important risk factors, including smoking, hyperlipidemia, hypertension, diabetes mellitus and positive family histories were elicited from all patients and controls. Family studies were not done, but detailed history-taking and clinical examination had excluded other causes of hypercoagulability, including diabetes, hypertension, liver disease, infections, malignancies, polycythemia, thrombocytosis, nephritic syndrome, use of oral contraceptives and hormone replacement therapy. Due to budgetary constraints, other thrombophilia markers such as serum homocysteine, factor V Leiden and raised factor VIII were not included.

### Sample collection and processing

All samples were collected from patients after stabilization for 10-12 weeks and none of the patients were on oral anticoagulants at the time of sampling. Fifty-two patients were not on any treatment, 20 were on low-dose aspirin and 13 were on coumarins, from which they were weaned off and put on low molecular weight heparin for three weeks before their samples were drawn for testing. Samples were collected by means of venipuncture with one-tenth volume of 0.109 M trisodium citrate. Platelet-poor plasma (count < 104/mm3) was prepared for testing APC-R by means of centrifugation at 200 g for 10 minutes, and followed by centrifugation at 10,000 g for 5 minutes. Plasma samples were stored as 10 aliquots of 200 µl in Eppendorf tubes at -40° Celsius until testing. The functional activities of protein C, protein S and AT were determined using kits from Diagnostica Stago, France (STA-Staclot for protein C, protein S and APC-R; STA deficient V for factor V; and STA-Stachrom for AT). The AT, protein C and protein S levels in test plasma were expressed as percentages (%) of the standard plasma. Fibrinogen estimation was done using Clauss's method.6 All abnormal results were reconfirmed on a fresh sample from the patients taken at least 12 weeks later. 

### Equipment and methods

All tests except for AT estimation were performed on a four-channel semiautomatic coagulometer (Diagnostica Stago, France). AT estimation was done on a biochemistry analyzer, as mentioned in a previous study on venous thrombosis.^7^ The diagnostic methods for detecting MI were troponin T, CK MB and aminotransferase; EKG or echocardiography during acute phase of MI followed by coronary angiography; radionuclide lung scanning or angiography for pulmonary embolism; arteriography for peripheral arterial occlusions; and CT scan, magnetic resonance imaging or arteriography for cerebral thrombosis. For a diagnosis of transient ischemic attack (TIA), a focal neurological deficit resolving within 24 hours was required, whereas if it persisted beyond 24 hours, a diagnosis of stroke was made. 

### Statistical analysis

The Epi Info 6 software was used for statistical analysis on the accrued data. The chi-square test was used for data analysis. P values were calculated and The Yates and Mantel-Haenszel corrections were made. The controls were compared with the patients in both disease subgroups. Odds ratios were calculated whenever possible. Fisher's exact test (both one and two-tailed) was performed and its results were used for drawing conclusions. The sample size was calculated considering a study power of 60%, odds ratio worth detecting of 1.00, exposure to risk factors among controls of 6% and a 90% confidence interval, resulting in a minimum sample of 53 patients.

## RESULTS

A total of 85 patients (M/F 78/7) and 50 age-matched controls (M/F 46/4) were studied. The mean ages and ranges for patients and controls were 37.2 years (3-45 years) and 34 years (5-45 years), respectively. The demographic data on the patients and controls are shown in Table 1. The means and ranges of values for seven quantitative thrombophilia markers are shown in Table 2. The normal levels of various parameters were derived from ranges obtained in control populations and did not vary significantly from the ranges mentioned in product inserts.

**Table 1:** Age and sex distribution of cases and controls and number of episodes in cases

	Mean age (range in years)	Sex Male/ Female	Single episode	Multiple episodes
Controls	34 (18-45)	46/4	NA	NA
Myocardial infarction	40.2 (27-53)	38/0	38	7*
Stroke	37.4 (3-49)	41/7	44	4

NA = not applicable

* Two patients were over 45 years at the time of recruitment, but had their first episode of MI before reaching 45 years age.

**Table 2:** Mean values and ranges for various parameters in controls, and comparison with values mentioned in product insert

Test	Range (controls)	Mean	Range or mean (product insert)
PT	11.1-13.2 s	12.1	11.5 -14.5
APTT	26-34 s	29	32.4
AT	79-124%	97	80-120
Protein C	89-150%	105	70-130
Protein S	91-150%	110	65-140
APC-R	120 - > 240 s	> 120	> 120 s
Fibrinogen	130-300 mg/dl	180	200-400
Lp(a)	10-23 mg/dl	15.1	10.7

PT = prothrombin time

APTT = activated partial thromboplastin time

**LA-PTT:** = lupus-sensitive APTT

AT = antithrombin

APCR = activated protein C resistance

Lp(a) = lipoprotein alpha

s = seconds

mg/dl = milligrams per deciliter.

Multiple episodes of thrombosis occurred in 11 patients, of whom seven had MI, three had stroke and one had both. A family history of stroke, MI or thrombophilia was present in 13 patients. The frequencies of thrombophilia markers and risk factors in patients and controls are shown in Table 3.

**Table 3:** Frequencies of thrombophilia markers and risk factors in controls and patients

Parameter No.	Thrombophilia marker or risk factor	Stroke (n = 48)	P value	MI (n = 37)	P-value	Control (n = 50)
1	No abnormality1	21	-	10	-	40
2	Protein S	5	0.06	3	0.42	1
3	Protein C	2	0.34	2	0.23	0
4	Antithrombin	2	0.97	4	0.06	0
5	APC-R	7	0.23	5	0.24	2
6	Fibrinogen	10	0.002	6	0.012	0
7	Lipoprotein(a)	5	0.86	14	0.09	5
8	Smoking	12	0.048	15	0.018	5
9	Dyslipidemia	3	0.78	7	0.045	2
10	Family history	4	0.01	7	0.005	0

**APC-R:** = activated protein C resistance

MI = myocardial infarction

Abnormal results for parameters 2-4 refer to low levels, while for parameters 6-9 they refer to increase in levels

1 = refers to parameters 2-9.

The mean fibrinogen levels for the controls and patients were 150 mg% and 279 mg% respectively, with a peak value of 1700 mg% in the latter. A cutoff of 500 mg/dl was taken as an indicator of raised fibrinogen, in order to make the diagnostic criteria more stringent. Abnormal low values for protein C, protein S and AT and raised values for fibrinogen and Lp(a) are presented in Table 4. Low factor V was present in a single case of MI and the levels were 51% of normal. This patient was only 27 years old and also had elevated Lp(a) (78 mg/dl) and raised fibrinogen (553 mg%). On echocardiography, he was found to have extensive anterior and lateral wall infarction. No risk factors could be identified in nearly 50% of the patients (15/31) who were negative for thrombophilia markers. A combination of thrombophilia markers and/or risk factors was seen in 36 of the remaining 54 patients (66.7%).

**Table 4:** Control mean versus patient mean with peak or lowest values

Parameter	Control	Stroke	MI
Protein C	105%	97.8 (62*)	103 (29*)
Protein S	110%	103 (27.4*)	109 (19*)
Antithrombin	97%	97.8 (62*)	95 (40*)
Factor V	103%	98%	114% (51*)
Lp(a)	21 mg/dl	37 (105†)	40 (105†)
Fibrinogen	180 mg/dl	400 (1150†)	389 (1700†)

MI = Myocardial infarction

* lowest value

† peak value. Any value below 120 seconds for modified activated partial thromboplastin time (APTT) was taken to be abnormal and indicative of activated protein C resistance (APC-R).

The clinical presentation was classical ischemic stroke in 37 cases, of which three had predominance of sensory symptoms, TIA in five cases and seizures in six patients. Classical features were present in 31/37 MI cases (83.8%): two each presented with unstable angina, non-Q and silent MI. Smoking was the commonest (40%) risk factor in this group, followed by dyslipidemia and a positive family history (19%).

### Statistically significant parameters

P values for various parameters are presented in Table 3. 

## DISCUSSION

The presence of a thrombophilia marker in 38.8% of the patients may appear a little high, but some other authors have reported the presence of a thrombophilia marker in 50% of young patients with MI with ≤ 1 risk factor.^2^ This can also be attributed to the marker selection, especially Lp(a) and fibrinogen. Contrary to the previous belief that factor V Leiden is rare in Indians,^8^ some recent studies have reported higher frequency of APC-R in Indian patients with venous thrombosis.^9^ ^-^ ^11^ In the present study, APC-R was detected in 14% of the patients and 4% of the controls. In a study by Khare et al., the prevalence of factor V Leiden was significantly higher in MI cases than in controls.^12^ The product insert mentions that there is excellent concordance with DNA analysis for factor V Leiden, so it is possible that most of the patients with APC-R would have shown positivity for factor V Leiden if DNA analysis had been done. The limitations of this study include the lack of DNA studies for detecting factor V Leiden, which is the commonest cause of APC-R. There is also a need to look for other mutations that could lead to APC-R in Indians.

None of the patients with deficiencies of AT, protein C or protein S had the abnormality in isolation, while APC-R was the sole abnormality in only 2/13 cases. This highlights the relevance of investigating multiple factors in combination as the cause of thrombotic episodes. Presence of antiphospholipid antibodies can cause the artifacts of low protein S and positive APC-R. The AT levels were in the range of 60-65% of normal activity in five out of the six patients and 40% in one patient. It is quite possible that these five would have yielded a normal value for AT level, thus showing the limited role of AT deficiency in arterial thrombosis.

In our study, low AT was found to be significantly related to MI, which is contrary to a previous report in the literature by Khare et al., who reported that protein C, protein S and AT deficiencies was seen in 3/120 cases each (2.5%).^12^ The fibrinogen levels in the present study were significantly elevated in MI patients, in agreement with previous Indian studies.^12^ ^-^ ^14^ Also in the present study, we observed that higher levels correlated with higher frequency of recurrence and severity of thrombotic episode. Lp(a) is a non-modifiable risk factor that has been shown to be an independent risk factor for thrombosis.^14^ ^-^ ^16^ Hence, both fibrinogen and Lp(a) must be evaluated in young patients with arterial thrombosis.

In our study, the AT levels were low in 6.5% of the patients, and were higher than in Indian studies on DVT cases.^11^ ^,^ ^12^ The AT levels were in the range of 60-65% of the normal range (80-130%) in 5/6 patients and it was 45% in one patient. It is possible that low AT levels acted on combination with other markers to cause thrombosis, but only moderate decreases in AT are associated with venous thrombosis.^17^ The patients and controls were also tested for antiphospholipid antibodies, including lupus anticoagulants and anticardiolipin antibodies, but these data have already been published and hence are not mentioned in this paper.

Thrombophilia screening is extremely expensive for a developing country like India. The combined commercial cost of functional assays for AT, factor V, protein C, protein S and APC-R is $ 300, while antigenic assays are four times as expensive and not easily available in this country. These tests should not be performed routinely and can be contemplated only if all other tests are negative. Furthermore, these tests must not be done during the acute stage. Testing for fibrinogen and Lp(a) is relatively inexpensive.

Morris et al. mentioned that testing for inherited thrombophilia is of no use in stroke cases of arterial origin.^18^ In a study on 129 young patients with MI, Celik et al. found that congenital thrombophilia did not contribute towards enhanced risk of disease.^19^ Ng et al. also concluded that the yield from performing extensive diagnostic tests is often poor and, in the majority of cases, no established curative measures exist.^20^

Concentrates of protein C, protein S and AT are extremely expensive and not available even in cases of severe deficiency, and so have a limited role in management. Therefore, extreme prudence has to be exercised in test selection and such tests must be done only after the patient has been stabilized and been taken off coumarin.

## CONCLUSION

Routine testing for fibrinogen could be useful in addition to well-recognized markers including AT, APC-R, protein S and protein C for all young patients with a primary thrombotic episode. There is an urgent requirement to study the Indian population for factor V Leiden and APC-R.
